# Changes in Production Parameters, Egg Qualities, Fecal Volatile Fatty Acids, Nutrient Digestibility, and Plasma Parameters in Laying Hens Exposed to Ambient Temperature

**DOI:** 10.3389/fvets.2020.00412

**Published:** 2020-07-17

**Authors:** Da-Hye Kim, Yoo-Kyung Lee, Sung-Dae Lee, Sang-Ho Kim, Sang-Rak Lee, Hong-Gu Lee, Kyung-Woo Lee

**Affiliations:** ^1^Department of Animal Science and Technology, College of Animal Science and Technology, Konkuk University, Seoul, South Korea; ^2^National Institute of Animal Science, Rural Development of Administration, Jeonju-si, South Korea

**Keywords:** heat stress, laying performance, fiber digestibility, volatile fatty acid, laying hens

## Abstract

The present study was undertaken to investigate the impact of heat stress on nutrient digestibility and tibia and reproductive traits, and changes in laying performance, egg qualities, fecal volatile fatty acids, and plasma parameters in laying hens. One-hundred twenty 52-week-old laying hens were raised in three temperature-controlled facilities with constant humidity (50% RH), either normal temperature (LT; 22°C) or heat stress considered being moderate (MT; 27°C) or severe (HT; 32°C) for 42 days. Feed intakes were consistently low (*p* < 0.01) in HT hens compared with those in LT or MT over the period of 42 days. Egg production kept markedly (*p* < 0.05) or numerically (*p* > 0.05) low in hens exposed to HT vs. LT or MT. Egg mass and egg weight were consistently low (*p* < 0.01) in hens exposed to HT compared with those raised under LT or MT. On the other hand, feed conversion ratio and frequency of dirty and cracked eggs were not significantly affected (*p* > 0.05) during the experimental period. HT-exposed hens consistently had lowered (*p* < 0.05) eggshell thickness and breaking strength, eggshell weight, and plasma Ca, P, and Mg levels compared with LT- or MT-treated hens. HT hens had lower (*p* < 0.01) relative oviduct weight and less number of large yellow follicles compared with those raised under LT or MT conditions at 42 days. Tibia traits measured at 42 days were not affected by any of heat treatments. Fecal volatile fatty acids tended to be higher in HT-exposed laying hens throughout the experiment. It was noted that digestibilities of neutral detergent fiber and dry matter were lowest (*p* < 0.05) in hens exposed to HT vs. LT or MT environments. Our study suggests that heat stress could lower laying performance, egg quality, and physiological parameters that are coupled with alterations in gut metabolites and mineral/lipid metabolism. The findings emerged from this study will help us design the nutritional and environmental strategies to mitigate the negative effect of heat stress on laying hens.

## Introduction

Under heat stress (HS) condition, chickens have very limited capacity to maintain homeostasis of body temperature via inherent physiological and behavioral changes (1) due to the feather coverage and lack of sweat glands. It is well-reported that HS lowers production parameters (2) including egg production, feed intake, and eggshell thickness, and impairs the endocrine system, acid-base imbalance (3), and reproductive organs' functions (4). In addition, HS increases the heterophil/lymphocyte ratio (3), an indicator of stress, and lowers the resistance of chickens to pathogens (5) by compromised gut integrity or alteration in gut microbiota shifting to less protective microflora. It is thus understood that HS lowers laying production, welfare, and health of laying hens leading to significant economic loss to the global industry.

Chickens use multiple physiological mechanisms to overcome the harmful effects of acute or chronic HS. Short-term HS (i.e., acute heat stress) promotes heat shock response (6) and alters gene expression (7). On the other hand, long-term HS (i.e., chronic heat stress) produces adaptation by changing the thermoregulatory activity (7). Thus, acute and chronic HS exhibits unharmonized body reactions in laying hens exposed to high ambient temperature. Corticosterone, a well-known stress hormone in chickens, is also induced in different ways depending on acute and chronic HS (2, 3). These contradictions suggest that physiological changes of laying hens are strongly influenced by both temperature and time exposed to HS.

Although the effect of HS, either acute or chronic, or direct or indirect, on laying hens has been reported (8, 9), there have been no HS reports, to our knowledge, that investigated time-dependent changes in productive and physiological responses over the course of HS. In addition, environment humidity, which is a triggering factor to HS (8), is often disregarded in many HS studies (8, 10) which prompted us to design different ambient temperatures with constant humidity in this study. Therefore, the main objective of the present study was aimed to reveal the effects of HS on the changes in production parameters, egg qualities, fecal volatile fatty acids, and plasma parameters during the course of HS treatment. Furthermore, the effect of HS on laying hen was also monitored by measuring the reproductive organ and tibia traits, and nutrient digestibility at the end of HS treatment. It is hoped that the results obtained in this study will help us understand the time-dependent changes in performance, host physiology, and egg quality by laying hens following HS exposure and will add to the existing scientific literatures to find the effective nutritional strategies to mitigate the negative HS impact on laying hens.

## Materials and Methods

### Bird Husbandry

One-hundred-twenty 52-week-old laying hens were housed in three environment chambers. Each chamber equipped with heater, air-conditioner, humidifier, de-humidifier, and controller, and had one-tier 20 cages, 1 m high from the floor. Each cage (41 × 37 × 40 cm; length × depth × height) had nipples and a trough feeder, and 2 hens per cage were housed. Two adjacent cages were considered a replicate and each hen had floor space of 759 cm^2^.

Hens were initially adapted to the chambers for 4 weeks at ambient temperature of 22°C and relative humidity (RH) of 50% and lighting program of 16 L/8 D. When they were 56 weeks of age, hens in each of the chambers received 1 of 3 different heat treatments for 42 days. In the first chamber, hens were continuously received 32°C and 50% RH, representing high HS treatment (HT). The second chamber was continuously exposed to 27°C and 50% RH, representing moderate HS (MT). Finally, hens in the third chamber were continuously exposed to 22°C and 50% RH, representing optimal temperature (LT). Temperature and humidity loggers (MHT-381SD; Lutron Electronic Enterprise Co., Taipei, Taiwan) were placed in each chamber. Initially, temperatures for MT and HT were chosen from a previous study (unpublished data). Hens in MT started to pant while those in HT exhibited moderate to severe behavioral changes including panting and wings spread. Heat increment was gradually increased (~2°C per 1 h) to reach the target temperature per chamber and kept constant throughout the experiment.

A corn-soybean meal-based commercial layer diet containing 13.9% CP, 0.53% available P, 3.28% Ca, and 2,620 kcal ME was used. Laying hens had *ad libitum* access to water and feed throughout the experimental period. Body weight per replicate was measured at fortnightly intervals and feed intake per replicate was measured at days 3, 7, and then weekly thereafter. Performance data were presented on a 2-week basis. In addition, early performance data during 7 days post HS treatment were indicated in the text when plausible.

### Egg Production and Quality

All eggs including intact, dirty, and broken eggs were daily collected and weighed. Hen-day egg production was calculated as (total number of eggs per cage/number of hens per cage) × 100. The percentage of dirty and broken eggs were calculated as (total number of dirty and broken eggs per cage/total number of eggs per cage) × 100. The feed conversion ratio (FCR) was calculated on a 2-week basis for each group in the study. The FCR was expressed as kg of feed consumed per kg of egg produced. The intact eggs (6 eggs/each replicate) collected preceding 3 consecutive days per replicate specified at days 3, 7, and weekly thereafter were used to measure egg qualities. Specific gravity of the egg was determined using the saline floatation method (11). The width and length of eggs were measured with caliper and used to calculate a shape index (12). Haugh unit, eggshell strength, eggshell thickness, and yolk color were recorded by digital egg tester DET-6000 (Navel, Kyoto, Japan). Freed yolk was weighed and measured for its width and length using caliper that used to calculate a yolk index (13). The residual albumen adhering to the eggshells was removed using an absorbent paper and dried in room temperature to determine eggshell weight.

### Plasma Parameters

Approximately 3 ml of blood per hen were drawn from the wing vein using heparinized syringes at days 1, 3, 7, 14, 21, 28, 35, and 42 following HS treatment. Plasma was separated by centrifugation at 200 g for 15 min and stored at −20°C until the analysis. Plasma samples were analyzed an automatic blood chemical analyzer (Film DRI CHEM 7000i, Fuji film, Tokyo, Japan). Individual plasma samples were analyzed for total cholesterol, triglyceride, high-density lipoprotein cholesterol (HDL cholesterol), calcium (Ca), Magnesium (Mg), and phosphorus (P). Nitric oxide (NO) in plasma was measured using modified Griess reagent (Sigma-Aldrich, St. Louis, MO, USA) and NO concentration was calculated from standard curve with sodium titrate.

### Organ and Tibia Characteristics

On day 42, one hen per replicate was euthanized by overdose of carbon dioxide for sampling liver, spleen, abdominal fat, ovary, oviduct, and tibia. Oviduct was weighed after removing eggs in the lumen. Large follicles over 0.5 cm were counted and recorded. Organ weights were expressed relative to live body weight. The width and length of the tibia were measured using a digital caliper. Tibia breaking strength was measured using an Instron (Model 3342, Instron Universal Testing Machine, Instron Corp., Norwood, MA, USA) with 50-kg load range with a crosshead speed of 50 mm/min with tibia supported on a 3.35-cm span. Tibias were dried at 102°C in a drying oven for 24 h and weighed for dry matter determination. The dried tibias were extracted with a Soxhlet apparatus (Soxtherm automatic, Gerhardt, Bonn, Germany) for 24 h to measure the fat-free tibias. The fat-freed tibias were then ashed at 600°C for 3 h and re-weighed.

### Fecal Nutrient Digestibility

During the last 3 days of the experiment, whole excreta samples per replicate were quantitively collected to determine the apparent total tract digestibility of dry matter (DM), crude protein (CP), crude fat, neutral detergent fiber (NDF), and crude ash. Fecal droppings per each replicate were collected four times daily stored at −20°C and pooled. Excreta samples were dried in a drying oven at 55°C for 72 h and ground for chemical analysis. Feed and excreta samples were analyzed in duplicate for DM, crude fat, CP, NDF, and crude ash as described elsewhere (14).

### Fecal Volatile Fatty Acid Analysis

Freshly void excreta samples exempted from uric acid and cecal droppings were sampled into sterilized tubes at fortnightly intervals and processed to measure volatile fatty acid (VFA) contents on the same day of sampling. Approximately 1 g of excreta was added in 9 ml of cold distilled water and homogenized using an ultra turrax (Digital Ultra-Turrax T25, IKA, Staufen, Germany). The mixture was added with 0.05 of saturated HgCl_2_, 1 ml of 25% H_3_PO_4_, and 0.2 ml of 2% pivalic acid and centrifuged at 1,000 × g at 4°C for 20 min. Then, the supernatant (1.5 ml) was collected and stored at −20°C before analysis. The concentrations of VFA in samples were measured using gas chromatography (6890 Series GC System, HP, Palo Alto, CA, USA) as described (15–17).

### Statistical Analysis

Two adjacent cages were considered an experimental unit. Before statistical analysis, the arcsine transformation was used for al percentage data. All data were analyzed using the general linear model (GLM) procedure of SAS (SAS Institute Inc, Cary, NC, USA). The results were presented as least square means and pooled standard error of the mean. Duncan's multiple range test was employed to determine means and differences among treatments. Significant differences among treatments were determined at probability of *p* < 0.05.

## Results

### Effects of HS on Body Weight Change and Feed Intake

Laying hens under MT and LT treatments slightly gained weights whereas those under HT treatment lost body weight during the experiment, leading to significant difference in weight loss (*p* < 0.05) in the HT group vs. MT and LT groups (Table 1). During the HS experiment, daily feed intake was significantly decreased in the HT group vs. MT and LT groups (*p* < 0.001; Table 1). No difference of daily feed intake was noted between MT and LT groups (Table 1) albeit that hens in MT vs. LT tended to consume less especially at days 28 and 42. Hens in HT group consumed 95.5–102.0 g per hen daily which were significantly decreased by ~18–22% and 13–20% compared with the LT and MT groups.

**Table 1:** The effect of heat stress on production performace^1^.

	**Temperature**, **°C**			**SEM^2^**	***P*-value**
	**22**	**27**	**32**		
**Body weight gain, g**					
Day 0–14	33.2^ab^	94.2^a^	−17.7^b^	21.29	<0.01
Day 14–28	30.5^a^	40.0^a^	−14.2^b^	12.32	<0.05
Day 28–42	19.6	4.2	19.8	8.48	0.35
Day 0–42	69.6^a^	138^a^	−12.1^b^	23.30	<0.05
**Feed intake, g/bird**	
Day 14	125.3^a^	126.4^a^	101.7^b^	3.59	<0.01
Day 28	130.4^a^	124.2^a^	102.0^b^	3.73	<0.01
Day 42	116.8^a^	109.9^a^	95.5^b^	2.82	<0.01
**Egg production, %**	
Day 14	80.7^a^	83.0^a^	66.5^b^	4.64	<0.05
Day 28	81.2	86.2	70.6	4.48	0.06
Day 42	81.6^a^	85.1^a^	65.9^b^	5.18	<0.05
**Feed conversion ratio, kg/kg**	
Day 14	1.83	1.89	1.88	0.10	0.91
Day 28	1.85	1.79	1.75	0.09	0.75
Day 42	1.67	1.59	1.89	0.15	0.33

1 *Values are least squares means representing 10 observations*.

2 *SEM, standard errors of the means*.

ab *Means with a different superscript differ (p < 0.05)*.

### Effects of HS on Laying Performance and Egg Quality

Hen-day egg production began to drop rapidly in the HT treatment group compared with the MT and LT groups (data not shown). As expected, HT-exposed hens had lowest egg production ranging from 66.5 to 70.6% which were significantly lower by on average 13–19% and 18–23% compared with the LT and MT groups. However, HS did not affect the percentages of dirty and cracked eggs at any age (*p* > 0.05; Table 2) and FCR (kg/kg; Table 1). Although not statistically significant, HT group had the highest FCR at day 42, being 13 and 19% higher, compared with the LT and MT groups.

**Table 2:** The effect of heat stress on dirty and cracked eggs, egg weight, and egg mass^1^.

	**Temperature**, **°C**			**SEM^2^**	***P*-value**
	**22**	**27**	**32**		
**Dirty and cracked eggs, %**					
Day 14	2.74	1.79	3.69	1.68	0.73
Day 28	2.26	3.45	1.90	1.09	0.58
Day 42	1.19	1.67	1.31	0.73	0.89
**Egg weight, g/egg**					
Day 14	64.7^a^	63.0^a^	59.0^b^	0.59	<0.01
Day 28	66.7^a^	66.3^a^	58.2^b^	1.73	<0.05
Day 42	63.9^a^	64.9^a^	56.2^b^	1.05	<0.01
**Egg mass, g/day**					
Day 14	52.3^a^	52.3^a^	39.3^b^	2.98	<0.05
Day 28	53.5^a^	57.0^a^	41.1^b^	2.82	<0.05
Day 42	52.5^a^	55.1^a^	37.5^b^	3.40	<0.05

1 *Values are least squares means representing 10 observations*.

2 *SEM, standard errors of the means*.

ab *Means with a different superscript differ (p < 0.05)*.

HS affected egg weight (Table 2). Under HS condition, egg weight was declined (*p* < 0.05) in the HT group compared with the LT and MT groups. Effect of HS on egg mass followed egg weight (Table 2), being the HT group *vs*. MT and LT groups maintained lowest at all specified days.

**Figure 1:**
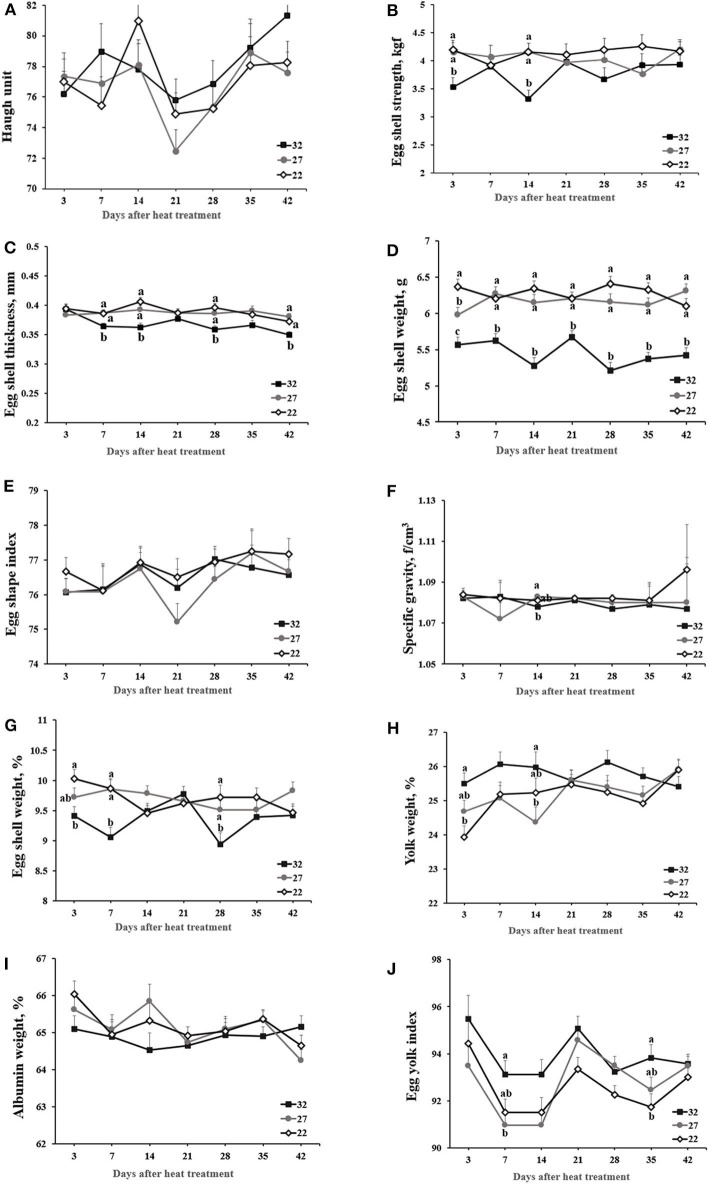
The effect of heat stress on egg quality. Laying hens were exposed to high (32°C), moderate (27°C), and optimal (22°C) ambient temperature for 42 days. Blood was obtained at the specified time points following heat treatment. Values with different superscripts differ significantly between treatments (*p* < 0.05). **(A)** Haugh unit. **(B)** Eggshell strength (kgf). **(C)** Eggshell thickness (mm). **(D)** Eggshell weight (g). **(E)** Egg shape index was calculated with the width and length of eggs. **(F)** Specific gravity (f/cm^3^). **(G)** Eggshell weight (%), **(H)** Yolk weight (%). **(I)** Albumin weight (%). **(J)** Eggyolk index was calculated with the width and length of yolks.

Haugh unit scores ranged between 72 and 82 and was not affected by heat treatment during 42-day experiment (Figure 1). Egg shape index was not affected. Specific gravity was not affected by heat treatment but declined in the HT vs. LT group at day 14. Eggshell traits (i.e., strength, thickness, and eggshell percentage) maintained low in the HT group compared with the MT and LT groups during the 42-day period. Of note, hens exposed to HT vs. LT exhibited a decline in eggshell strength at days 3 and 14, eggshell weight at days 3, 7, and 28, and eggshell thickness at days 7, 14, 28, and 42. HT- vs. LT-exposed hens maintained higher percentage of yolk weight, but had lower percentage of albumin weight.

### Effects of HS on Plasma Biochemical Parameters

**Figure 2:**
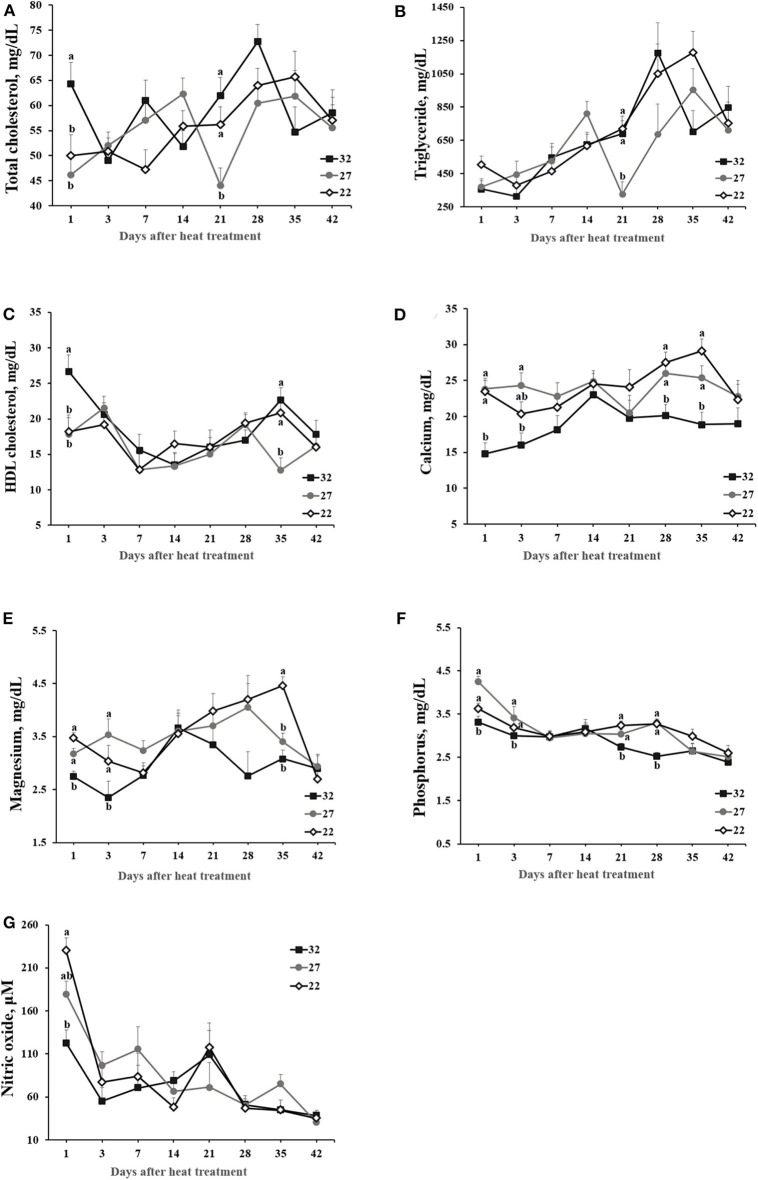
The effect of heat stress on plasma parameters. Laying hens were exposed to high (32°C), moderate (27°C), and optimal (22°C) ambient temperature for 42 days. Blood was obtained at the specified time points following heat treatment. Values with different superscripts at specific time point differ significantly between treatments (*p* < 0.05). **(A)** Total cholesterol (mg/dL). **(B)** Triglycerides (mg/dL). **(C)** High density lipoprotein (HDL) cholesterol (mg/dL). **(D)** Calcium (mg/dL). **(E)** Magnesium (mg/dL). **(F)** Phosphorus (mg/dL). **(G)** Nitric oxide (μM).

HS affected concentrations of total cholesterol, triglyceride, HDL cholesterol, Mg, P, and nitric oxide (Figure 2). Although HT rapidly increased the concentrations of total cholesterol and HDL cholesterol (*p* < 0.05) at day 1 post heat treatment (*p* < 0.05), no apparent kinetic patterns depending on the heat treatments were detected thereafter. Triglyceride tended to increase with age, but the concentration was not clearly different between heat treatments over the course of experiment. In general, the concentrations of minerals (i.e., Ca, P, and Mg) maintained low in HT- vs. LT- or MT-exposed hens although statistical difference was not detected at all ages. Nitric oxide tended to decline with age, but HT only caused to rapidly drop nitric oxide in plasma samples compared with MT and LT groups at day 1 post HS treatment (*p* < 0.05; Figure 2).

### Effects of HS on Characteristics of Internal Organs

HS affected the relative weights of spleen (*p* = 0.06) and oviduct (*p* < 0.05) at 42 days (Table 3). Relative spleen weight was lowest in the HT group compared with the MT and LT groups, but the difference was not statistically significant. HT vs. MT and LT treatments significantly lowered oviduct weight and number of large yellow follicles. On the other hand, HS treatment did not affect the relative weights of liver, ovary, and abdominal fat. Tibia characteristics including fat-free DM weight and ash weight were not affected by HS treatment for 42 days (Table 4).

**Table 3:** The effect of heat stress on relative organ weights (g/100 g of live body weight) and number of follicles^1^.

	**Temperature**, **°C**			**SEM^2^**	***P*-value**
	**22**	**27**	**32**		
Liver	2.74	3.36	3.08	0.24	0.20
Spleen	0.11	0.10	0.09	0.01	0.06
Ovary	0.40	0.48	0.42	0.04	0.27
Oviduct	3.95^a^	3.67^a^	3.09^b^	0.15	<0.05
Abdominal fat	4.80	3.81	3.36	1.01	0.59
Large yellow follicles	5.20^a^	5.40^a^	4.30^b^	0.27	<0.05

1 *Values are least squares means representing 10 observations*.

2 *SEM, standard errors of the means*.

ab *Means with a different superscript differ (p < 0.05)*.

**Table 4:** The effect of heat stress on tibia characteristics^1^.

	**Temperature**, **°C**			**SEM^2^**	***P*-value**
	**22**	**27**	**32**		
Fresh weight, g/100 g of live body weight	0.65	0.62	0.70	0.02	0.07
Length, cm	11.7	11.5	11.5	0.13	0.49
Width, cm	0.32	0.30	0.31	0.01	0.35
Strength, kgf	24.7	21.9	24.1	1.84	0.53
Dry matter, %	50.8	50.7	47.6	1.55	0.27
Fat-free dry matter, g	6.36	6.05	6.01	0.27	0.62
Ash, g	3.47	3.25	3.36	0.16	0.61
Ash/fat-free dry matter, %	55.0	53.8	55.9	1.57	0.64

1 *Values are least squares means representing 10 observations*.

2 *SEM, standard errors of the means*.

### Effect of HS on Concentration of Fecal Volatile Fatty Acids

Volatile fatty acids in fresh fecal samples were measured fortnightly intervals (Table 5). At day 14, acetate was elevated (*p* = 0.11) by 1.86- and 1.67-fold in the HT and MT groups compared with the LT group. Propionate was highest when hens were exposed to HT vs. MT and LT (*p* < 0.05). At day 28, propionate and butyrate were significantly higher (*p* < 0.05) in the MT group compared with the LT and HT groups. At day 42, acetate and total volatile fatty acids were elevated in the HT group compared with the LT and MT groups. Propionate kept low in the MT group vs. HT and LT groups, but the difference was not statistically significant.

**Table 5:** The effect of heat stress on concentration of fecal volatile fatty acid (mM/g)^1^.

	**Temperature**, **°C**			**SEM^3^**	***P*-value**
	**22**	**27**	**32**		
**Day 14**					
Acetate	24.4	40.7	45.4	7.18	0.11
Propionate	1.27^b^	1.19^b^	2.35^a^	0.12	<0.05
Butyrate	1.42	1.31	2.64	0.53	0.29
Total VFA^2^	33.3	43.2	54.0	7.97	0.65
**Day 28**					
Acetate	26.6	27.6	26.8	3.74	0.99
Propionate	2.10^b^	2.35^a^	1.69^b^	0.38	<0.05
Butyrate	1.71^b^	2.97^a^	1.28^b^	0.51	<0.05
Total VFA	32.2	35.3	29.8	3.62	0.88
**Day 42**					
Acetate	28.1^b^	39.2^b^	63.5^a^	5.59	<0.01
Propionate	4.06	1.13	4.79	1.08	0.07
Butyrate	3.66	2.24	3.91	0.85	0.13
Total VFA	36.9^b^	42.6^b^	72.2^a^	7.55	<0.05

1 *Values are least squares means representing 10 observations*.

2 *Total VFA, volatile fatty acid*.

3 *SEM, standard errors of the means*.

ab *Means with a different superscript differ (p < 0.05)*.

### Effect of HS on Nutrient Digestibility

HS influenced nutrient digestibility at 42 days (Table 6). HT vs. MT and LT lowered the digestibility of dry matter (*p* < 0.05), crude fat (*p* = 0.05), and NDF (*p* < 0.05) in laying hens. Crude ash digestibility gradually decreased (*p* = 0.14) as the HS treatment increased. On the other hand, HS treatment did not affect crude protein digestibility.

**Table 6:** The effect of heat stress on nutrient digestibility at day 42^1^.

	**Temperature**, **°C**			**SEM^2^**	***P*-value**
	**22**	**27**	**32**		
Dry matter, %	74.3^a^	72.5^a^	64.5^b^	2.19	<0.05
Crude protein, %	67.2	67.4	69.3	2.48	0.83
Crude fat, %	74.8	65.2	60.4	3.82	0.05
Neutral detergent fiber, %	52.7^a^	47.5^a^	37.0^b^	2.33	<0.05
Crude ash, %	48.4	40.1	36.4	3.79	0.14

1 *Values are least squares means representing 10 observations*.

2 *SEM, standard errors of the means*.

ab *Means with a different superscript differ (p < 0.05)*.

## Discussion

Laying hens are susceptible to high ambient environment due to the lack of sweat glands, limited water evaporation through respiratory systems, and high heat production associated with enhanced egg production (18). Multiple behavioral or physiological mechanisms are used to restore homeostasis disrupted by HS throughout the course of heat exposure and the consequences of HS will be reflected in laying performance, egg and eggshell qualities, blood parameters, nutrient digestibility, and metabolic profiles of gut microbiota in laying hens. In this study, we monitored the changes in production and physiological indices of laying hens exposed to HS treatment for 42 days. As the current study focused on kinetic patterns of the variables over the course of different HS treatments, age effect was not considered in the statistical analysis. Mortality kept low between HS treatments although HT regime had higher mortality (LT = 2, MT = 1; HT = 5).

### Effects of HS on Laying Performance and Egg Qualities

As expected, HT vs. LT and MT caused to lose body weight which corroborates with previous studies (4, 9). The observed loss in body weight noted in our study may be in part due to HS-induced decrease in feed consumption. Indeed, feed intake was significantly lowered in HT group compared with LT and MT groups, of which findings agree with earlier reports (4, 5, 19). It is however pointed out that body weight loss for 42 days was considered minimal indicating that HS employed in this study was not dramatic thus enabling laying hens to maintain homeostasis by lowering feed intake. In addition to feed intake, hen-day egg production, egg weight, and egg mass were negatively associated with high ambient temperature. Exposure of laying hens to high ambient temperature caused reproductive deficiency as seen by a reduction in reproductive activities and egg quality. In line with our finding, a significant decrease in relative oviduct weight of HT-exposed hens was noted indicating the impaired efficiency of reproductive performance (4).

Our study clearly explains compensatory response of hens exposed to high ambient temperature by limiting feed intake and reduction in feed intake *per se* is mainly responsible for impaired laying performance. The latter statement is supported by unaltered feed conversion ratio during the experimental period. However, delayed response of feed conversion ratio to HT vs. LT and MT groups indicates that decline in feed intake and laying performance as one of early heat-stress protective mechanisms may not be sufficient to overcome the negative effect of HS *per se* on host metabolism in the prolonged HS treatment. It has been reported that HS *per se* impaired gut integrity, gut microflora, and host immunity in laying hens (20), which might play in part a role in HT-induced depression in laying performance. It is of note that FCR tended to be high in HT *vs*. MT and LT groups at 42 days post HS treatment. This result was due to the observed low feed intake with gradual decline in egg production in HT group. This result further highlights that reproductive performance is gradually, but not dramatically, declined in laying hens exposed to HS.

Exposure of hens to high ambience temperature resulted in a significant decrease in eggshell qualities including eggshell weight, eggshell thickness, and eggshell strength. Eggs laid from HT-exposed laying hens were significantly lighter compared with those from MT and LT groups. These results agree with those of Castro et al. (21) who found that high ambience temperatures decreased egg weight. These results are associated with a reduction in feed consumption, led to a decrease in eggshell and albumen weights. In contrast, relative weight of egg yolk was heavier in HT vs. LT group, indicating that decreased egg weight is associated with alteration in eggshell as reported elsewhere (8, 9). Clear deterioration in eggshell quality indicates the negative effect of HS on mineral metabolism. When birds are exposed to high temperature, they increase respiration rate (i.e., panting) to maximize evaporation (i.e., latent heat) due to the lack of sweating gland. This causes to reduce blood CO_2_ and ${\text{HCO}}_{3}^{-}$ and elevates blood pH (19). In addition, Hu et al. (22) reported that plasma Ca level was decreased in laying hens exposed to HS. Thus, it is likely that limited supply via blood vessel for calcium and bicarbonate to form eggshell at the oviduct leads to poor eggshell quality. Over the course of HS, concentrations of Ca, P, and Mg in plasma samples were kept low which supports the negative effect of HS on eggshell formation.

### Effects of HS on Plasma Biochemical Parameters

Biochemical and endocrine changes are considered among the critical host responses to nutritional and environmental stimuli. The concentrations of TCHO, TG, and HDL cholesterol in plasma samples can be used as physiological indicators of stress during biochemical and endocrine changes (23). Chickens under HS release glucocorticoids which in turn mobilize lipids from adipose tissue to liver in laying hens (24). In this study, concentrations of TCHO and HDL cholesterol were significantly elevated in laying hens exposed to HT vs. MT and LT at day 1 upon HS treatment. This confirms the function of HDL which is to transport cholesterol to liver from body tissues (23). The elevated concentration of HDL cholesterol in HT-exposed laying hens in this study corroborates with previous findings (23, 25) that HDL cholesterol was elevated in chickens exposed to HS. In contrast, lipid parameters (e.g., TCHO and HDL cholesterol) returned to normal after 1 day of HS treatment indicating the capability of laying hens in maintaining lipid metabolism. The latter finding indicates that laying hens are able to endogenously synthesize cholesterol and TG in liver to meet host's demand (e.g., cell renewal and egg yolk).

NO is produced by chicken monocytes and macrophage upon exposure to enteric pathogens or stressors and considered an innate immunity (26). NO acts a vasodilator signaling blood vessel to relax enabling efficient nutrient transport upon ingestion or to target organs and is produced from L-arginine by NO synthase (27). Thus, early inhibition of NO production in HT vs. MT and LT groups is likely from sudden decrease in arginine availability due to the decreased feed intake and/or impaired absorption of arginine. The latter was reported elsewhere (28) in HS-exposed chickens that decreased absorption of arginine.

The concentrations of Mg, P, and Ca were consistently low in HT *vs*. MT and LT groups in this study. As the sources of these minerals are from diet origin, the reduced concentrations of plasma minerals are likely due to the reduced feed intake or impaired nutrient digestion and absorption. Although statistical significance was not reached, we found that the digestibility of crude ash was lowered by on average 24.8% in HT vs. LT groups. Collectively, these findings explain the HT-induced deterioration in eggshell quality including breaking strength and relative eggshell weight.

### Effects of HS on the Characteristics of Internal Organs

In this study, laying hens exposed to HT vs. MT and LT groups had decreased relative weights of spleen and oviduct, and the number of large yellow follicles. On the other hand, HS did not affect tibia characteristics including breaking strength and fat-free ash DM contents. These results indicate that HS has more negative effects on reproductive organs than Ca storage organs, which explains the HT-induced decrease in laying production in this study. HS is known to lower plasma gonadal steroid hormone (29) and estradiol concentration and expression of receptors for luteinizing hormones (9) resulting in oviduct regression. In addition, it has been reported that reduced ovarian functions by HS may be in part secondary to diverting blood flow to surface to dissipate heat production (8). In contrast to earlier findings that HS could affect the tibia characteristics (30), no significant effect was noted in this study. Our study is in line with that of Castro et al. (21), indicating that mineral turnover and metabolism in tibia seemed to be affected minimally, if present, by the HS regime used in this experiment. It should be kept in mind that we used the 32°C for HT treatment which is considered moderate in HS experiments. In this study, HT vs. MT and LT tended to lower the relative spleen weight. Although we did not measure immune indices for acquired immune responses or subpopulations of splenocytes, atrophied spleen by HS may indicate impaired immune competence as reported (4). It has been well-known that HS impaired innate and cell-mediated immunities in chickens (31).

### Effect of HS on Concentration of Fecal Volatile Fatty Acids and Nutrient Digestibility

The VFAs are of bacteria-mediated metabolites and can be used to assess the indicator of bacterial fermentation in chickens (32, 33). The VFAs such as acetate, propionate and butyrate are among the major metabolites from fermentation by gut commensals on undigested carbohydrates (32) while branched-chain fatty acids are on undigested protein in the lower tract (33). Exposure of laying hens to high temperatures significantly increased the amount of acetate and total VFA in excreta samples. This finding could be due to the increase in the availability of substrates for distal gut microflora. It has been reported that HS disrupted the balance of gut microflora and lowered digestibility of nutrients such as fiber in chickens (34, 35). In this study, digestibility of NDF was significantly impaired in laying hens exposed to HT vs. MT and LT. It is well-known that HS damages gut integrity in chickens and inhibits the activities of host digestive enzymes (36). Thus, increased concentrations of VFAs in HT groups noted in this study are likely due to increased distally available substrates for gut microflora. Our finding suggests that exogenous enzymes acting on water-soluble non-starch polysaccharides might help chicken overcome HS-induced digestive disorder which needs to be clarified.

## Conclusion

HS clearly lowers feed intake, egg production, and eggshell quality. The concentrations of Ca, P, and Mg in plasma samples were kept low in HT vs. MT and LT groups. Parameters relating to lipid metabolism were altered upon early HS exposure. Finally, HS lowered digestibility of dry matter, crude ash, and NDF at 42 days. Collectively, our study shows that laying hens exposed to HT, but not MT, lowered laying performance and eggshell qualities via lowered feed intake coupled with alteration in gut microbiome and mineral metabolism. Currently, behavioral and physiological factors including immune response, antioxidant defense, and stress indicators (e.g., corticosterone, heterophil:lymphocyte ratio) are under investigation in our laboratory that will elucidate the adaptive mechanisms of laying hens exposed to ambient temperature.

## Data Availability Statement

All datasets presented in this study are included in the article/supplementary material.

## Ethics Statement

The animal study was reviewed and approved by Institutional Animal Care and Use Committee at Konkuk University (KU18149).

## Author Contributions

D-HK and K-WL contribution to conception and design, interpretation of data, acquisition of data, and analysis and drafting of the manuscript. Y-KL, S-DL, S-HK, S-RL, and H-GL revising the manuscript critically. All authors have read and contributed to the final manuscript.

## Conflict of Interest

The authors declare that the research was conducted in the absence of any commercial or financial relationships that could be construed as a potential conflict of interest.

## References

[1] Wu QJ Lin N Wu XH Wang GY Lin L Glutamine alleviates heat stress-induced impairment of intestinal morphology, intestinal inflammatory response, and barrier integrity in broilers. Poult Sci. (2018) 97:2675–83. 10.3382/ps/pey123 29788452

[2] Zhang P Yan T Wang X Kuang S Xiao Y Lu W Probiotic mixture ameliorates heat stress of laying hens by enhancing intestinal barrier function and improving gut microbiota. Ital J Anim Sci. (2017) 16:292–300. 10.1080/1828051X.2016.1264261

[3] He SP Arowolo MA Medrano RF Li S Yu QF Chen JY Impact of heat stress and nutritional interventions on poultry production. Worlds Poult Sci J. (2018) 74:647–64. 10.1017/S0043933918000727

[4] Attia YA El AEHEA Abedalla AA Berika MA Al-Harthi MA Kucuk O Laying performance, digestibility and plasma hormones in laying hens exposed to chronic heat stress as affected by betaine, vitamin C, and/or vitamin E supplementation. Springerplus. (2016) 5:1619 10.1186/s40064-016-3304-0 27652192 PMC5028346

[5] Alhenaky A Abdelqader A Abuajamieh M Al-Fataftah AR. The effect of heat stress on intestinal integrity and Salmonella invasion in broiler birds. J Therm Biol. (2017) 70:9–14. 10.1016/j.jtherbio.2017.10.015 29108563

[6] Wang WC Yan FF Hu JY Amen OA Cheng HW. Supplementation of Bacillus subtilis-based probiotic reduces heat stress-related behaviors and inflammatory response in broiler chickens. J Anim Sci. (2018) 96:1654–66. 10.1093/jas/sky092 29528406 PMC6140875

[7] Jiang S Mohammed AA Jacobs JA Cramer TA Cheng HW. Effect of synbiotics on thyroid hormones, intestinal histomorphology, and heat shock protein 70 expression in broiler chickens reared under cyclic heat stress. Poult Sci. (2020) 99:142–50. 10.3382/ps/pez571 32416795 PMC7587863

[8] Rostagno MH. Effects of heat stress on the gut health of poultry. J Anim Sci. (2020) 98:skaa090. 10.1093/jas/skaa090 32206781 PMC7323259

[9] Nawab A Ibtisham F Li G Kieser B Wu J Liu W. Heat stress in poultry production: mitigation strategies to overcome the future challenges facing the global poultry industry. J Therm Biol. (2018) 78:131–9. 10.1016/j.jtherbio.2018.08.010 30509629

[10] Li WX Chen YQ Zhao LH Ma QG Zhang JY Ji C. No copper supplementation in a corn-soybean basal diet has no adverse effects on late-phase laying hens under normal and cyclic high temperatures. Poult Sci. (2018) 97:1352–60. 10.3382/ps/pex447 29452393

[11] Balevi T Coskun B. Effects of dietary copper on production and egg cholesterol content in laying hens. Br Poult Sci. (2004) 45:530–34. 10.1080/00071660412331286253 15484729

[12] Mónus F Barta Z Repeatability analysis of egg shape in a wild tree sparrow (*Passer montanus*) population: a sensitive method for egg shape description. Acta Zool Academ Sci Hung. (2005) 51:151–62.

[13] Keener KM McAvoy KC Foegeding JB Curtis PA Anderson KE Osborne JA. Effect of testing temperature on internal egg quality measurements. Poult Sci. (2006) 85:550–55. 10.1093/ps/85.3.550 16553288

[14] Official Methods of Analysis of the Association of Official Analytical Chemists 18th ed Arlington, VA: Association of Official Analytical Chemists (2005).

[15] van der Wielen PW Biesterveld S Notermans S Hofstra H Urlings BA van Knapen F. Role of volatile fatty acids in development of the cecal microflora in broiler chickens during growth. Appl Environ Microbiol. (2000) 66:2536–40. 10.1128/AEM.66.6.2536-2540.2000 10831435 PMC110578

[16] Meimandipour A Shuhaimi M Hair-Bejo M Azhar K Kabeir BM Rasti B. *In vitro* fermentation of broiler cecal content: the role of lactobacilli and pH value on the composition of microbiota and end products fermentation. Lett Appl Microbiol. (2009) 49:415–20. 10.1111/j.1472-765X.2009.02674.x 19725887

[17] Kim DH Han SM Keum MC Lee S An BK Lee S-R. Evaluation of bee venom as a novel feed additive in fast-growing broilers. Br Poult Sci. (2018) 59:435–42. 10.1080/00071668.2018.1476675 29774758

[18] Khan RU Naz S Dhama K Chromium: pharmacological applications in heat stressed poultry. Int J Pharmacol. (2014) 10:213–317. 10.3923/ijp.2014.213.217

[19] Barrett NW Rowland K Schmidt CJ Lamont SJ Rothschild MF Ashwell CM. Effects of acute and chronic heat stress on the performance, egg quality, body temperature, and blood gas parameters of laying hens. Poult Sci. (2019) 98:6684–92. 10.3382/ps/pez541 31573614 PMC8914008

[20] Varasteh S Braber S Akbari P Garssen J Fink-Gremmels J. Differences in susceptibility to heat stress along the chicken intestine and the protective effects of galacto-oligosaccharides. PLoS One. (2015) 10:e0138975. 10.1371/journal.pone.0138975 26402906 PMC4581695

[21] Castro FLS Kim HY Hong YG Kim WK. The effect of total sulfur amino acid levels on growth performance, egg quality, and bone metabolism in laying hens subjected to high environmental temperature. Poult Sci. (2019) 10:4982–93. 10.3382/ps/pez275 31152669

[22] Hu JY Hester PY Makagon MM Xiong Y Gates RS Cheng HW. Effect of cooled perches on performance, plumage condition, and foot health of caged White Leghorn hens exposed to cyclic heat. Poult Sci. (2019) 98:2705–18. 10.3382/ps/pez039 30796446

[23] Odihambo Mumma J Thaxton JP Vizzier-Thaxton Y Dodson WL. Physiological stress in laying hens. Poult Sci. (2006) 85:761–69. 10.1093/ps/85.4.761 16615361

[24] Sahin R Küçük O. A simple way to reduce heat stress in laying hens as judged by egg laying, body weight gain and biochemical parameters. Acta Vet Hung. (2001) 49:421–30. 10.1556/004.49.2001.4.6 11942121

[25] Puvadolpirod S Thaxton JP Model of physiological stress in chickens 1. Response parameters. Poult Sci. (2000) 79:363–69. 10.1093/ps/79.3.363 10735203

[26] Sundaresan NR Saxena VK Sastry KVH Anish D Saxena M Nagarajan K. Nitric oxide: a possible mediator of ovulation and postovulatory follicle regression in chicken. Anim Reprod Sci. (2007) 101:351–57. 10.1016/j.anireprosci.2007.01.011 17306940

[27] Khajali F Wideman RF. Dietary arginine: Metabolic, environmental, immunological and physiological interrelationships. Worlds Poult Sci J. (2010) 66:751–66. 10.1017/S0043933910000711

[28] Saeed M Abbas G Alagawany M Kamboh AA El-Hack MEA Khafaga AF. Heat stress management in poultry farms: a comprehensive overview. J Therm Biol. (2019) 84:414–25. 10.1016/j.jtherbio.2019.07.025 31466781

[29] Henriksen R Groothuis TG Rettenbacher S. Elevated plasma corticosterone decreases yolk testosterone and progesterone in chickens: linking maternal stress and hormone-mediated maternal effects. PLoS One. (2011) 6:e23824. 10.1371/journal.pone.0023824 21886826 PMC3160319

[30] Vakili R Rashidi AS Sobjanirad S Effects of dietary fat, vitamin E and zinc supplementation on tibia breaking strength in female broilers under heat stress. Afr J Agric Res. (2010) 5:3151–56. 10.5897/AJAR10.248

[31] Khan RU Naz S Nikousefat Z Tufarelli V Javadani M Rana N Effect of vitamin E in heat-stressed poultry. Worlds Poult Sci J. (2011) 67:469–78. 10.1017/S0043933911000511

[32] Peng Q Zeng XF Zhu JL Wang S Liu XT Hou CL. Effects of dietary *Lactobacillus plantarum* B1 on growth performance, intestinal microbiota, and short chain fatty acid profiles in broiler chickens. Poult Sci. (2016) 95:893–900. 10.3382/ps/pev435 26772658

[33] Qaisrani SN Moquet PCA van Krimpen MM Kwakkel RP Verstegen MWA Hendriks WH. Protein source and dietary structure influence growth performance, gut morphology, and hindgut fermentation characteristics in broilers. Poult Sci. (2014) 93:3053–64. 10.3382/ps.2014-04091 25306462

[34] Sahin K Sahin N Onderci M. Vitamin E supplementation can alleviate negative effects of heat stress on egg production, egg quality, digestibility of nutrients and egg yolk mineral concentrations of Japanese quails. Res Vet Sci. (2002) 73:307–12. 10.1016/S0034-5288(02)00126-1 12443690

[35] Wang S Mahfuz S Song H Effects of flammulinavelutipes stem base on microflora and volatile fatty acids in caecum of growing layers under heat stress condition. Rev Bras Cienc Avic. (2019) 21: eRBCA-2019-0989 10.1590/1806-9061-2019-0989

[36] Khan RU Naz S Nikousefat Z Selvaggi M Laudadio V Tufarelli V Effect of ascorbic acid in heat-stressed poultry. Worlds Poult Sci J. (2002) 68:477–90. 10.1017/S004393391200058X

